# Motif-guided sparse decomposition of gene expression data for regulatory module identification

**DOI:** 10.1186/1471-2105-12-82

**Published:** 2011-03-22

**Authors:** Ting Gong, Jianhua Xuan, Li Chen, Rebecca B Riggins, Huai Li, Eric P Hoffman, Robert Clarke, Yue Wang

**Affiliations:** 1 Bradley Department of Electrical and Computer Engineering, Virginia Tech, Arlington, VA 22203, USA; 2 Lombardi Comprehensive Cancer Center and Department of Oncology, Physiology and Biophysics, Georgetown University, Washington, DC 20057, USA; 3 Bioinformatics Unit, RRB, National Institute on Aging, National Institutes of Health, Baltimore, MD 21224, USA; 4 Research Center for Genetic Medicine, Children's National Medical Center, Washington, DC 20010, USA

## Abstract

**Background:**

Genes work coordinately as gene modules or gene networks. Various computational approaches have been proposed to find gene modules based on gene expression data; for example, gene clustering is a popular method for grouping genes with similar gene expression patterns. However, traditional gene clustering often yields unsatisfactory results for regulatory module identification because the resulting gene clusters are co-expressed but not necessarily co-regulated.

**Results:**

We propose a novel approach, motif-guided sparse decomposition (mSD), to identify gene regulatory modules by integrating gene expression data and DNA sequence motif information. The mSD approach is implemented as a two-step algorithm comprising estimates of (1) transcription factor activity and (2) the strength of the predicted gene regulation event(s). Specifically, a motif-guided clustering method is first developed to estimate the transcription factor activity of a gene module; sparse component analysis is then applied to estimate the regulation strength, and so predict the target genes of the transcription factors. The mSD approach was first tested for its improved performance in finding regulatory modules using simulated and real yeast data, revealing functionally distinct gene modules enriched with biologically validated transcription factors. We then demonstrated the efficacy of the mSD approach on breast cancer cell line data and uncovered several important gene regulatory modules related to endocrine therapy of breast cancer.

**Conclusion:**

We have developed a new integrated strategy, namely motif-guided sparse decomposition (mSD) of gene expression data, for regulatory module identification. The mSD method features a novel motif-guided clustering method for transcription factor activity estimation by finding a balance between co-regulation and co-expression. The mSD method further utilizes a sparse decomposition method for regulation strength estimation. The experimental results show that such a motif-guided strategy can provide context-specific regulatory modules in both yeast and breast cancer studies.

## Background

Transcriptional gene regulation is a complex process that uses a network of interactions to [1]. A central problem remains the accurate identification of transcriptional modules or gene sub-networks involved in the regulation of critical biological processes [2]. For cancer research, these sub-networks can help provide a signature of the disease that is potentially useful for diagnosis, or suggests novel targets for drug intervention. The biomedical research literature and several specific databases contain sequence information, gene expression profiling data, and small scale biological experiments that allow investigators to reconstruct gene regulatory networks and explore the direct effects of transcription factors on gene expression.

Recently, the bioinformatics community has explored various computational approaches for transcriptional module identification [3-7]. These approaches can be classified into two major categories. The first category uses clustering methods to explore the similarity in gene expression patterns to form gene modules. The second approach uses projection methods to infer latent (hidden) components with which to group genes into modules. A growing literature documents attempts to reconstruct gene networks by applying clustering methods [8,9] and their more sophisticated variants such as statistical regression [10] and Bayesian networks [11]. While this line of work is important to help formulate hypotheses, there are many limitations on using clustering methods for regulatory module inference. One common challenge is detecting the interactions between transcription factors and their target genes based on gene expression data alone. For regulatory module identification, it is critical to distinguish 'co-regulation' from 'co-expression', and to understand the relationship between co-regulation and co-expression. Generally, genes with highly homologous regulatory sequences (co-regulation) should have a similar expression pattern (co-expression). However, the reverse is likely not true; co-expressed genes must not necessarily exhibit common regulatory sequences [12]. Traditional clustering analysis often returns clusters lacking shared regulatory sequences, thus making the biological relevance of these clusters relatively low for the identification of regulatory mechanisms.

A group of projection methods from the second category, including principle component analysis (PCA), independent component analysis (ICA), and non-negative matrix factorization (NMF) [13-15], have also been extensively applied for transcriptional module identification. These methods decompose gene expression data into components that are constrained to be mutually uncorrelated or independent, and then cluster genes based on their loading in the components. Since these methods do not cluster genes based on their expression similarity, they are better equipped to find co-regulated gene modules. One major difficulty using such projection approaches is that the components usually represent the joint effects of many underlying transcription factors. Thus, the components do not correspond to individual known transcription factors (TFs), making the biological interpretation of the components very difficult.

To overcome the above-mentioned shortcomings, several integrative methods have been proposed that integrate TF-gene interaction data with gene expression data. For instance, network component analysis (NCA) has been recently developed to successfully estimate the TF activities of regulatory networks using both ChIP-on-chip and gene expression data [16]. Note that NCA heavily relies on ChIP-on-chip data for network connectivity information with which to define regulatory modules. Thus, the NCA scheme is not readily applicable to many biological studies where adequate network connectivity information is not available (due to lack of adequate ChIP-on-chip data). To deal with this difficulty, Sabatti and James [17] were among the first to use motif information as the initial network topology, subsequently adopting a Bayesian algorithm to reconstruct regulatory modules. While theoretically elegant, this approach needs to estimate the posterior probability, a joint distribution of network topology and transcription factor activity. Even using the Gibbs sampling technique, it is a formidable task to estimate the joint distribution when the number of samples is limited.

We now propose a novel approach, namely motif-guided sparse decomposition (mSD), to identify co-regulated transcriptional modules by integrating motif information and gene expression data. The mSD method is a Bayesian-principled method without the need to estimate the joint distribution. Instead, a two-step approach is used to first estimate transcription factor activity and then regulation strength on the target genes. A motif-guided clustering method is developed to help estimate transcription factor activity by taking into account both co-expression and co-regulation. A sparse decomposition step is then applied to estimate the regulation strength of predicted regulatory networks. To evaluate the performance of the proposed approach, we applied the mSD method to simulated and real yeast cell cycle data, showing an improved performance in identifying three kinds of coherent modules associated with known cell cycle transcription factors. We then applied our approach to a molecular profiling study of estrogen dependence in breast cancer cells, with the goal of recovering condition-specific transcriptional modules related to estrogen action. The results demonstrated that our approach effectively finds important condition-specific regulatory modules that are functionally relevant to estrogen signaling pathways.

## Methods

**Figure 1:**
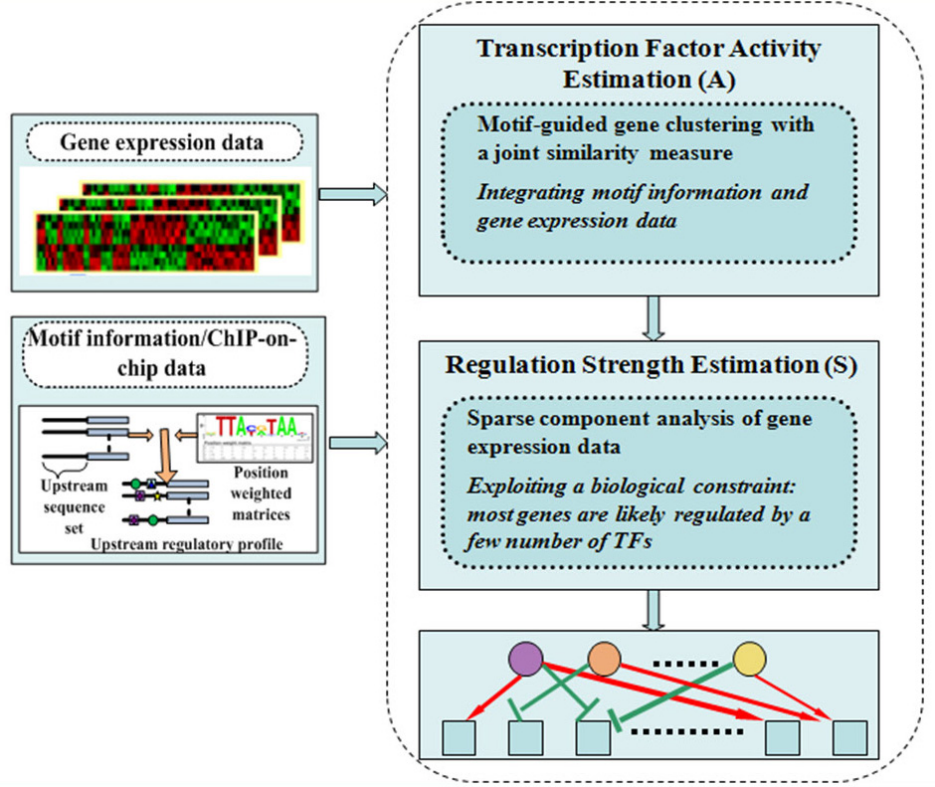
**A block diagram of the motif-guided sparse decomposition (mSD) approach**. The mSD approach consists of the following two steps: (1) transcription factor activity estimation by motif-guided clustering and (2) regulation strength estimation by sparse decomposition.

The overall scheme of the proposed mSD approach is illustrated in Figure 1. We start by extracting motif information from upstream DNA sequences of genes, followed by a two-stage approach to integrate motif information and gene expression data for regulatory module identification. In the first stage, we use a motif-guided clustering method for transcription factor activity estimation by maximizing the motif support for co-expressed gene modules. In the second stage, we use a sparse decomposition method for regulation strength estimation to enforce that the genes in a module are likely regulated by a few transcription factors. Finally, regulatory modules are reconstructed from the detected active regulators and their target genes that exhibit large regulation strengths. In this section, we will give a detailed description of each major component in the mSD approach. Note that the mSD software package is implemented and made available at http://www.cbil.ece.vt.edu/software.htm.

### Latent variable model

We adopt a latent variable model that has been used in Liao *et al*. [16] and Kao *et al*. [18] to establish a link between gene expression data and motif information. The central theme of the model is that gene expression measurements can be largely determined by the unknown activities of transcription factors acting on known binding motifs (TFs). Using log-ratios of gene expression measurements, a simplified, yet biologically justified, linear model can be formulated as follows [16]:(1)

where *x_pg _*is defined as the logarithm of the expression ratio of gene *g *between data sample *p *and control sample, *a_pt _*the activity level of TF *t *in sample *p *and *s_tg _*the regulation strength of TF *t *onto gene *g*. The log-ratios of gene expression **X **∈ **R**^*m*×*N *^,(*N *>> 1) are expressed as a linear combination of log-ratios of TF activity (**A **∈ **R**^*m*×*n *^) weighted by their regulation strength (**S **∈ **R***^n×N ^*). Note that *m *is the number of samples, *N *is the number of genes, and *n *is the number of TFs.

In general, the number of TFs is much smaller than the number of transcribed genes (*n *<<*N*) and most genes are regulated only by a small number of TFs. Hence, the matrix **S **that describes the regulation strength between the TFs and their regulated genes is sparse. Further, the number of TFs (*n*) is usually greater than the number of samples (*m*), i.e., *n *>*m *, such that Equation (1) represents an underdetermined linear system (ULS). To obtain a sparse solution to this ULS, we develop a two-stage approach to estimate transcription factor activity (**A) **and regulation strength (**S) **sequentially.

### Transcription factor activity estimation

A generic approach for transcription factor activity estimation is to use a clustering method to find representative genes whose expression profiles (columns of **X) **can be utilized to estimate **A **[19]. For a theoretical justification of the identifiability of **A**, please refer to Section 1.1 in the supplementary material. Many clustering techniques have been proposed to cluster gene expression data, such as *k*-means clustering [20] and self-organizing maps [21], which are designed to find gene expression patterns by grouping genes with similar expression profiles. Very recently, an affinity propagation (AP) algorithm has been proposed for data clustering that shows an improved performance [22]. Based on an *ad hoc *pair-wise similarity function between data points, AP seeks to identify each cluster by one of its elements, the so-called *exemplar*. AP takes as input a collection of real-valued similarities between data points, where the similarity *s*(*i*, *k*) indicates how well data point *k *is suited to be the exemplar for data point *i*. The goal is to maximize the similarity *s*(*i*, *k*) or equivalently, to minimize the Euclidean distance [22], *d*(*i*, *k*) = ||**x***_i _*- **x***_k_*||^2^, where **x***_*i *_*and **x***_*k *_*are two column vectors of gene *i *and gene *k*, respectively, in **X**.

However, direct application of the AP clustering technique to gene expression data will only give rise to co-expressed gene clusters. To identify gene regulatory modules, we need a clustering technique to integrate motif information and gene expression data, aiming to find co-regulated gene clusters with co-expressed patterns. We here propose a motif-guided clustering method to find a group of genes that not only is of similar expression pattern but also shares a common set of binding motifs as much as possible.

### Motif-guided gene clustering with a joint similarity measure

To incorporate motif information, we propose a new similarity measure, taking into account both expression similarity and motif binding similarity, for the AP clustering method. The motif information can be represented by a TF-gene binding strength matrix, **W **= [*w*(*t*, *g*)], considering a set of *n *TFs binding onto a set of *N *genes. Each element of **W**, i.e., *w*(*t*, *g*), denotes the binding strength of TF *t *onto gene *g*. As a common practice, the binding strength is usually approximated by a position weight matrix (PWM) that contains log-odds weights for computing a match score between a binding site and an input DNA sequence [23]. For a detailed description of how to generate the binding strength matrix, please refer to Section S1.2 in the supplementary material of this paper. Given the binding strength of TF *t* onto gene *i *(*w*(*t*, *i*)) and that of TF *t *onto gene *k *(*w*(*t *, *k*)), the joint binding strength of TF *t *onto both gene *i *and gene *k *is proportional to *w*(*t*, *i*)×*w*(*t*, *k*), assuming that these two binding events are independent. Thus, for all possible TFs (TF *t*, *t *= 1,..., *n*) binding onto gene *i *and gene *k*, it is reasonable to use the sum of their joint binding strengths to measure the likelihood of gene *i *and gene *k *being co-regulated by the possible set of TFs (TF *t*, *t *= 1,..., *n*):(2)

For motif-guided clustering, we propose the following pair-wise similarity measure to simultaneously consider the binding motif likelihood and gene expression similarity:(3)

where *λ *is a trade-off parameter that controls the contribution from two different information sources: motif information and gene expression data. When incorporated into an AP clustering method, the first term in Eq. (3) is used to find a group of genes with similar expression pattern, while the second term estimates those genes that should share a common set of TFs.

Ideally, the clustering result will generate a better representation of the transcription factor activity that underlies a co-regulated group of genes. However, both motif information and gene expression data are noisy because the binding motif is a very short DNA sequence [24] and there is often a low signal-to-noise ratio in gene expression measurements [25]. The impact of the noises can be clearly observed in two extreme cases: (1) the gene cluster resulting from (noisy) motif information alone will show a noisy expression pattern; (2) the cluster resulting from gene expression data alone will often gain little support in terms of being regulated by a shared set of motifs. Therefore, it is important to understand the contribution of each data source and assign its proper weight. The trade-off parameter λ in Eq. (3) is used to alleviate the effects of noise. In the following section, we will design an entropy-based measure, in conjunction with a non-uniformity measure, to help find the optimal value for the trade-off parameter λ.

### Determination of the trade-off parameter

To measure the relative contribution of motif information to gene clustering, we propose an entropy-based measure to capture the property that a regulatory module should be regulated by a unique set of active transcription factors. For each gene cluster, an enrichment analysis is first performed to identify the significant motifs associated with the genes in the cluster. Specifically, a hyper-geometric test is designed to calculate the significance value (*p*-value) of a motif (motif *t*) enriched in the cluster. The testing procedure can be described as follows. The null distribution is generated by randomly sampling the entire gene population (with *N *genes) as many times as possible (approximately 10,000 times) to form random gene clusters. Let us assume that the gene cluster *j *under examination consists of *N_j _*genes in which *N_b _*genes have the support of motif *t*, while in the entire gene population the total number of genes that contain the motif t in their promoters is *N_B_*. For the randomly generated clusters (each with a size of *N_j_*), we count the number of genes containing motif *t *in each cluster, denoted as *i_r_*, to finally form the null distribution. The *p*-value for motif *t *enriched in cluster *j *can then be calculated as follows:(4)

With the *p*-value for each motif's enrichment, we calculate the motif emission frequency [26] for all the motifs in each cluster. For a particular cluster index *j*, *j *= 1,..., *J*, a set of motif frequencies can be defined as ***θ***_*j *_= (*θ*_*j*1_, *θ*_*j*2_,..., *θ*_*jn*_), where ***θ***_*jt *_= -log_10 _*p*_*jt*_, *t *= 1,..., *n *and *p_jt _*is the *p*-value obtained from Eq. (4). We then normalize ***θ***_*j *_by  to ensure that each element in ***θ***_*j *_falls in the range of [0, 1]. Treating motif occupancy as a random variable associated with an appropriate probability space, we can quantitatively measure the 'uncertainty' of motif occupancy in cluster *j*, from an information-theoretic perspective, by the following entropy definition [27]:(5)

The entropy is then normalized to be in the range of [0, 1] as divided by the maximum entropy (*H_max _*(***θ***_*j*_)), i.e., ; the maximum entropy is acheived when the motif occupancy is uniformly distributed. Summing over all the clusters, we can obtain the mean entropy to measure the overall 'uncertainty' of motif occupancy in the clusters as follows:(6)

Conceptually, when motifs are randomly distributed (with an assumed uniform distribution) among the clusters, the mean entropy reaches its maximum; conversely, when motifs are uniquely distributed for each cluster (cluster-specific), the mean entropy reaches its minimum.

To measure the relative contribution of gene expression data to gene clustering, we adopt a non-uniformity measure [28] to characterize the co-expression nature of the genes in a cluster. The non-uniformity of expression pattern is measured as inversely proportional to the variance of gene expression weighted by an appropriate weighting factor as shown in the following equation:(7)

Where  is the variance of gene expression pattern for cluster *j*(*j *= 1,..., *j*),  the maximum variance for all clusters, and *w_j _*is the weight of cluster *j *defined as the proportion of genes to the entire gene population.

By varying the trade-off parameter λ in Eq. (3), the AP clustering method will generate different clustering results. This outcome is predictable because both the motif information and gene expression data are noisy and will affect the clustering results. Particularly, when λ is small, the contribution from gene expression data dominates, which will give rise to gene clusters with small non-uniformity of expression pattern but large entropy of motif occupancy (not cluster-specific). In contrast, when λ is large, the contribution from motif information dominates, leading to gene clusters with large non-uniformity but small entropy of motif occupancy (cluster-specific). Therefore, it is important to find the optimal λ value to alleviate the noise impact on finding regulatory modules. We propose to use the following cost function to combine the measure of motif occupancy (Eq. (6)) and that of expression pattern (Eq. (7)) as follows:(8)

Theoretically, the cost function *C*(λ) is a U-shaped function; when λ reaches its optimal value, the cost function *C*(λ) reaches its minimum. In other words, by minimizing *C*(λ) we can find the optimal value of λ to take advantage of both the motif information and gene expression data, while alleviating the noise impact on gene clustering.

We can extend this cost function to a weighted form by using a trade-off parameter *μ*: *C*(*μ, λ*) = *μH*(*λ*)*+*(1*-μ*)*NonU*(*λ*), where 0 *≤ μ ≤ *1. By controlling *μ *we can obtain different sets of gene clusters with different degrees of motif occupancy and similarity in expression pattern. To determine an appropriate parameter λ, we use a simplified version of the cost function *C*(*μ*, *λ*): *C*(*λ*) *= H*(*λ*)*+NonU*(*λ*) (which is equivalent to the case of *μ *= 0.5), to help find an appropriate balance between motif occupancy and expression pattern for regualtory module identification. A simplified assumption here is that it is equally important to consider both co-regulation (measured by the entropy for motif occupancy) and co-expression (measured by non-uniformity of expression pattern) for regulatory module identification. Nevertheless, we use *C*(*μ*, *λ*) to examine the robustness of parameter *λ *for the microarray data analyzed in this paper, ensuring that the selected parameter *λ *is not sensitive to a particular choice of parameter *μ*.

### Regulation strength estimation

We use the sparse component analysis (SCA) approach [19] to exploit a well-known biological constraint that most genes are likely regulated by a few transcription factors, and then to estimate the regulation strength matrix **S**. Specifically, we have devised a projected "active subspace" algorithm for regulation strength estimation that can be described as follows:

(1) Initialize source **S **with a matrix **W**, which comes from either Chip-on-chip data or TF-gene binding strength matrix searched from TRANSFAC [29].

#### Loop

(2) Iterate for every column of **S **(which is corresponding to each gene)

a. If sparseness constraints on the current column of **S **(denote **s***_g_*) apply, project **s***_g _*to be desired sparse by making its *L*_1 _norm larger than a predefined sparseness threshold, while having the *L*_2 _norm unchanged. (For the definition of sparseness, please refer to [30].)

b. In the projected space, detect approximately which TFs are "active"; the term "active" is used to refer to the TFs with "considerably nonzero" strengths.

c. We assume that the first *q *TFs, {*s_tg_*}, *t *= 1,... *q*, have been found to be *inactive*. Find the new estimation of **s***_g _*by minimizing the cost function  subject to **x***_g _*= **As**_*g*_.

#### Until convergence

Notice that a major step in the above algorithm (Step (2a)) requires a projection operator that enforces sparseness by explicitly setting both *L*_1 _and *L*_2 _norms. This operator, fortunately, has been found by Hoyer [30] to incorporate sparseness constraint in the context of non-negative matrix factorization (NMF). We use this projection operator in the SCA approach to find the closest (in the Euclidean sense) sparse vector **s***_g _*with a desired *L*_1 _and *L*_2 _norm. The cost function in Step (2c) is designed to minimize the regulation strength of "inactive" TFs, while letting the regulation strength of "active" TFs to change freely in order to fulfil the imposed constraint **x***_g _*= **As***_g _*. This can also be viewed as a form of projection into an active subspace [31], resulting in an elegant mathematical approach to obtain the solution to a Karush-Kuhn-Tucker (KKT) system (for more details, please see Section S2 in the supplementary material).

## Results and Discussion

### Synthetic and real yeast data

To validate the proposed integrative approach, we applied mSD to synthetic and real yeast cell cycle data for regulatory module identification, and then compared its performance with those of other approaches including FastNCA [32] and sparse decomposition [19]. For the synthetic data set, we used a network generator, *SynTReN *[33], to produce a benchmark gene expression data set based on a synthetic *S*. *cerevisiae *transcriptional regulatory network. *SynTReN *generated 15 samples of expression data with a set of 345 genes in different conditions. The genome-wide location data (ChIP-on-chip data) [7] were then used to provide the binding information and these data were integrated with the gene expression data to extract transcription factor activity and estimate regulation strength.

To evaluate the performance of the mSD approach, we compared its performance with those of other similar methods, including FastNCA [32] and sparse decomposition (SD) [19]. Performances were measured by Receiver Operating Characteristic (ROC) analysis and the area under the ROC curve (AUC). The ROC curve measures the sensitivity and specificity of a method by calculating true-positive (TP) rate against false-positive (FP) rate. To generate a ROC curve, we first ranked the target genes for each TF according to their connection strengths in **S**, and then we calculated the true and false positive rates by running down the ranked gene list one at a time. To investigate the impact of noise on the respective performances of mSD and FastNCA, the binding information was obtained from the ChIP-on-chip data with different cut-off *p*-values (0.01, 0.05 and 0.1); a large cut-off *p*-value results in a high false positive rate in binding information (a high noise level).

In this experiment, we selected the following 11 well known regulatory TFs: ARG80, DAL82, GCN4, GCR2, HAP1, MIG1, RGT1, RTG1, RTG3, STE12 and XBP1, to calculate the averaged TP rates and FP rates for ROC analysis. Additional file 1, Figure S1 shows the ROC curves of three different approaches and Table 1 summarizes the AUCs of the ROC curves. For more analysis results, please refer to Additional file 1, Figure S2, Additional file 1, Figure S3 and Additional file 1, Table S1 in the supplementary material, which show detailed performance information on gene module identification for several transcription factors. As can be seen from the figures and tables, the mSD approach outperforms the other two methods in identifying co-regulated genes in all three cut-off *p*-values. Surprisingly, the performance of FastNCA is worse than that of SD even though no binding information is used in the SD approach. However, FastNCA largely depends on correct network topology, assuming noiseless binding information. When the noise level in binding information is relatively large, the performance of FastNCA degrades to an unacceptable degree. In contrast, the mSD approach finds a subset of target genes to reinforce the consistency between binding information and gene expression data, limiting the noise impact from both binding information and gene expression data.

**Table 1:** AUCs of mSD, SD and FastNCA methods, respectively, under different cut-off *p*-values

	mSD	SD	Fast NCA
cut-off *p*-value = 0.1	**0.7160**	0.6912	0.5707
cut-off *p*-value = 0.05	**0.7799**	0.6881	0.5891
cut-off *p*-value = 0.01	**0.8024**	0.6801	0.5547

To further evaluate our algorithm, we applied the mSD approach to a cell cycle data set obtained under the condition of arrest of a *cdc*15 temperature-sensitive mutant [34]. As a pre-processing step, we employed KNNimpute [35] to fill in missing values and then identified 800 cell cycle-related genes as the gene subpopulation to test the mSD approach. For the mSD approach, we set the trade-off parameter λ in Eq. (3) as 0.08 for this experiment, since the cost function, *C*(λ) (Eq. (8)), reached its minimum at λ = 0.08 (see Additional file 1, Figure S4 in the supplementary material for the *C*(λ) curve). The modified cost function *C*(*μ*, *λ*) can also be found in Additional file 1, Figure S5 in the supplementary material, which supports the robustness of the selected parameter *λ *with respect to parameter *μ*. Since there is no ground truth of target genes available for this experiment, we used the functional enrichment of regulatory modules to compare the performance of mSD with that of another method, COGRIM [36]. COGRIM is derived from a Bayesian hierarchical model and implemented using the Gibbs sampling technique. COGRIM can help infer the activation or inhibition of TFs acting on their target genes, with an integration of microarray gene expression data, ChIP-on-chip data, and motif information. The top GO enrichment *p*-values were transformed to negative logarithm values and averaged over all identified modules. The averaged enrichment score for the mSD method is 3.900, which is slightly better than the score for COGRIM (3.894), demonstrating that the mSD method can help identify functionally coherent gene clusters associated with specific TFs.

### Breast cancer cell line data

We then applied the mSD approach to breast cancer cell line data to help understand estrogen signaling and action in breast cancer cells. Greater than 70% of invasive breast cancers diagnosed each year in the U.S. express detectable levels of estrogen receptor alpha (ER, ER+) [37]. The most potent natural ligand for ER is 17β-estradiol, which can regulate the proliferation of breast cancer cells and alter their cytoarchitectural and phenotypic properties [37,38]. Antiestrogens, such as Tamoxifen and Fulvestrant, are widely used in the treatment of these breast cancers and they produce a significant survival benefit for some patients. However, half of these cancers will recur, and recurrent metastatic breast cancer remains an incurable disease. It is, therefore, clinically and biologically important to understand what transcriptional programs regulate these recurrence events [39,40].

To gain insights into the transcriptional programs that drive tumor recurrence, we have collected and acquired breast cancer cell line data in estrogen-induced and estrogen-deprived conditions, respectively. The estrogen induced data set is a time course microarray data set obtained from the ER+, estrogen-dependent breast cancer cell line MCF-7, treated with 17β-estradiol (E2) [41]. The estrogen-deprived data set consists of a series of breast cancer variants that closely reflect clinical phenotypes of endocrine sensitive tumors [39]. The breast cancer variants are also derived from the MCF-7 cell line, including MIII cells and LCC1 cells. MIII cells were derived directly from MCF-7 and became estrogen independent and proliferate aggressively after six months of selection *in vivo *in ovariectomized athymic mice. LCC1 cells were derived from MIII following further selection *in vivo*. Both cell lines remain ER+ and exhibit an estrogen-independent but antiestrogen sensitive phenotype [39,40].

We focused on twenty six breast cancer and estrogen receptor (ER) related transcription factors, which are listed in Table 2. This set of key transcription factors were previously identified and known to be involved in the estrogen receptor signaling (AP-1, CREB, ERα, NFκB, STATs [42]); authentic *cis *binding sites in breast cancer cell lines (C/EBP, Forkhead [43]); or overexpressed in estrogen receptor (ER)-positive breast tumors (EGR-1 [44,45], ETF [46], MYB [47], p53 [48]). Meanwhile, we also included some motifs involved in cell cycle or apoptosis (MYC/MAX [49], NFY [50], PBX1[51]). For each identified TF, a position weight matrix (PWM) was chosen from the vertebrate non-redundant profiles within the TRANSFAC database [29]. Further motif information was obtained from published ChIP-on-chip experiments [43], and we generated a final list of twenty six transcription factors (Table 2).

**Table 2:** Twenty six breast cancer and ER-related transcription factors

V$AP1_Q2_01	V$AP1_Q4_01	V$CREBP1CJUN_01	V$CEBP_Q3	V$CEBPA_01
V$CEBPGAMMA_Q6	V$CREB_02	V$CREB_Q3	V$CREB_Q2	V$NFKB_Q6_01
V$SP1_Q6	V$ER_Q6	V$ETF_Q6	V$MYCMAX_03	V$STAT_Q6
V$STAT_01	V$EGR1_01	V$FOXJ2_02	V$FOXP1_01	V$MYB_Q3
V$P53_02	V$PBX_Q3	V$PBX1_03	V$NFY_Q6_01	V$NFY_01
V$CEBPDELTA_Q6				

The motif information was obtained from the TRANSFAC database [29] and ChIP-on-chip experiments [43]. All human promoter DNA sequences were obtained from the UCSC Genome database [52]; we searched 5,000 bp upstream from the transcription start site (TSS). With all vertebrate position weight matrices (PWMs) provided by the TRANSFAC 11.1 Professional Database [29], the Match™ [53] algorithm was used to generate a gene-motif binding strength matrix with cut offs that minimize the false-positive rate.

**Figure 2:**
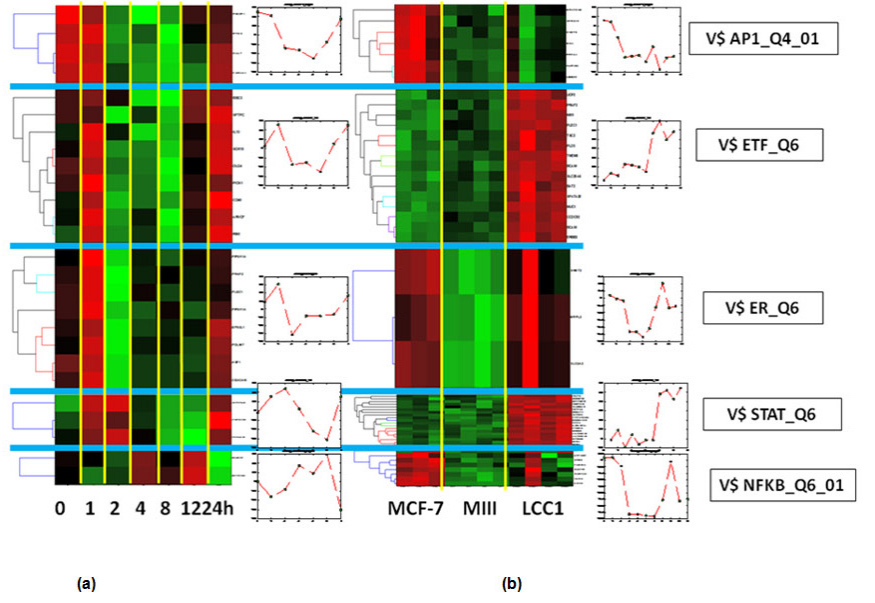
**Transcription factor activity estimated by the mSD approach**. (a) Estimated activities of the five transcription factors (AP1, ETF, ER, STAT and NFκB) in estrogen-induced condition. (b) Estimated activities of the five transcription factor bind sites in estrogen-deprived condition.

For the mSD approach, we optimized the trade-off parameter *λ *in Eq. (3) by examining the cost function *C*(λ) (Eq. (8)) (see Additional file 1, Figure S6 in the supplementary material for the detailed *C*(λ) curves). As shown in Additional file 1, Figure S7 in the supplementary material, the selected parameter *λ *is robust against the parameter *μ *in the modified cost function *C*(*μ*, *λ*). With the mSD approach to integrate motif information and gene expression data, we identified several key regulatory networks associated with estrogen signaling. Figure 2 shows the activities of five transcription factors (AP1, ETF, ER, STAT, NFκB) in estrogen-induced and estrogen-deprived conditions, respectively, that exhibit distinctive patterns of regulation. Transcription factor activities clearly show different actions in response to estrogen induction (Figure 2(a)). V$AP1_Q4_01 was activated within 1 hour after estrogen treatment; V$ETF_Q6 and V$ER_Q6 were also activated early, but showed a subsequent decrease in activity followed by a second activation event by 24 hours; V$STAT_Q6 exhibited a response to estrogen induction within 2 hours. This STAT activity estimation correlates well with previous findings that STATs are activated via the tyrosine phosphorylation cascade after ligand binding and stimulation of the cytokine receptor-kinase complex [54]. One of the mechanisms by which ER signaling occurs involves protein-protein interactions; activated estrogen receptors interact directly with transcription factors such as nuclear factor κB (NFκB), activator protein-1 (AP-1), and specificity protein-1 (SP1), to activate gene transcription [55]. As shown in Figure 2(a), an extended period of NFκB activation can be observed from 4 hours to 12 hours, which could be explained, at least in part, by such a mechanism.

Figure 2(b) shows the activities of these five transcription factors in the estrogen-deprived condition. Activation of ER can be clearly observed in LCC1 cells, along with activation of both ETF (V$ETF_Q6) and STAT (V$STAT_Q6), suggesting that the additional *in vivo *selection has led to further adaptations in ER signaling in these cells. To understand the mechanisms behind this, we examined both transcript factor activity (**A**) and regulation strength (**S**) to gain some insights into condition-specific regulation programs, particularly, the program in the estrogen-deprived condition for ETF and STAT. For example, we examined the target genes of EGFR-regulating transcription factor ETF (HUGO gene symbol: TEAD2, V$ETF_Q6) to understand its regulatory role in estrogen-deprived condition; ETF is known to stimulate EGFR transcription and might play a role in the overexpression of this growth factor receptor [46]. As expected, there is a large overlap between the identified ETF target gene sets in the two conditions, which are listed in Table 3 (see the supplementary material, Additional file 2, for the target genes of the other four TFs (AP1, ER, STAT, NFκB)). These genes are enriched in the following Gene Ontology terms: 'cell adhesion', 'cell cycle process', 'negative regulation of progression through cell cycle', 'regulation of kinase activity' and 'regulation of transferase activity and apoptosis'.

**Table 3:** Target genes of ETF (V$ETF_Q6) in both E2-induced and ER-deprived conditions

Probe Set ID	GENE_SYMBOL	Gene Name
200646_S_AT	NUCB1	NUCLEOBINDIN 1
200690_AT	HSPA9	HEAT SHOCK 70 KDA PROTEIN 9B (MORTALIN-2)
201373_AT	PLEC1	PLECTIN 1, INTERMEDIATE FILAMENT BINDING PROTEIN 500 KDA
201573_S_AT	ETF1	EUKARYOTIC TRANSLATION TERMINATION FACTOR 1
201601_X_AT	IFITM1	INTERFERON INDUCED TRANSMEMBRANE PROTEIN 1 (9-27)
201753_S_AT	ADD3	ADDUCIN 3 (GAMMA)
201842_S_AT	EFEMP1	EGF-CONTAINING FIBULIN-LIKE EXTRACELLULAR MATRIX PROTEIN 1
201910_AT	FARP1	FERM, RHOGEF (ARHGEF) AND PLECKSTRIN DOMAIN PROTEIN 1 (CHONDROCYTE-DERIVED)
201984_S_AT	EGFR	EPIDERMAL GROWTH FACTOR RECEPTOR (ERYTHROBLASTIC LEUKEMIA VIRAL (V-ERB-B) ONCOGENE HOMOLOG, AVIAN)
202088_AT	SLC39A6	SOLUTE CARRIER FAMILY 39 (ZINC TRANSPORTER), MEMBER 6
202235_AT	SLC16A1	SOLUTE CARRIER FAMILY 16 (MONOCARBOXYLIC ACID TRANSPORTERS), MEMBER 1
202295_S_AT	CTSH	CATHEPSIN H
202304_AT	FNDC3A	FIBRONECTIN TYPE III DOMAIN CONTAINING 3A
202429_S_AT	PPP3CA	PROTEIN PHOSPHATASE 3 (FORMERLY 2B), CATALYTIC SUBUNIT, ALPHA ISOFORM (CALCINEURIN A ALPHA)
202602_S_AT	HTATSF1	HIV-1 TAT SPECIFIC FACTOR 1
202730_S_AT	PDCD4	PROGRAMMED CELL DEATH 4 (NEOPLASTIC TRANSFORMATION INHIBITOR)
202826_AT	SPINT1	SERINE PEPTIDASE INHIBITOR, KUNITZ TYPE 1
202979_S_AT	CREBZF	HCF-BINDING TRANSCRIPTION FACTOR ZHANGFEI
203079_S_AT	CUL2	CULLIN 2
203278_S_AT	PHF21A	PHD FINGER PROTEIN 21A
203358_S_AT	EZH2	ENHANCER OF ZESTE HOMOLOG 2 (DROSOPHILA)
203456_AT	PRAF2	PRA1 DOMAIN FAMILY, MEMBER 2
203493_S_AT	CEP57	CENTROSOMAL PROTEIN 57 KDA
203607_AT	INPP5F	INOSITOL POLYPHOSPHATE-5-PHOSPHATASE F
203855_AT	WDR47	WD REPEAT DOMAIN 47
203869_AT	USP46	UBIQUITIN SPECIFIC PEPTIDASE 46
204129_AT	BCL9	B-CELL CLL/LYMPHOMA 9
204527_AT	MYO5A	MYOSIN VA (HEAVY POLYPEPTIDE 12, MYOXIN)
204629_AT	PARVB	PARVIN, BETA
204710_S_AT	WIPI2	WD REPEAT DOMAIN, PHOSPHOINOSITIDE INTERACTING 2
204989_S_AT	ITGB4	INTEGRIN, BETA 4
204995_AT	CDK5R1	CYCLIN-DEPENDENT KINASE 5, REGULATORY SUBUNIT 1 (P35)
205222_AT	EHHADH	ENOYL-COENZYME A, HYDRATASE/3-HYDROXYACYL COENZYME A DEHYDROGENASE
205258_AT	INHBB	INHIBIN, BETA B (ACTIVIN AB BETA POLYPEPTIDE)
206231_AT	KCNN1	POTASSIUM INTERMEDIATE/SMALL CONDUCTANCE CALCIUM- ACTIVATED CHANNEL, SUBFAMILY N, MEMBER 1
206574_S_AT	PTP4A3	PROTEIN TYROSINE PHOSPHATASE TYPE IVA, MEMBER 3
206604_AT	OVOL1	OVO-LIKE 1(DROSOPHILA)
207038_AT	SLC16A6	SOLUTE CARRIER FAMILY 16 (MONOCARBOXYLIC ACID TRANSPORTERS), MEMBER 6
207844_AT	IL13	INTERLEUKIN 13
208296_X_AT	TNFAIP8	TUMOR NECROSIS FACTOR, ALPHA-INDUCED PROTEIN 8
208754_S_AT	NAP1L1	NUCLEOSOME ASSEMBLY PROTEIN 1-LIKE 1
208876_S_AT	PAK2	P21 (CDKN1A)-ACTIVATED KINASE 2
209135_AT	ASPH	ASPARTATE BETA-HYDROXYLASE
209241_X_AT	MINK1	MISSHAPEN-LIKE KINASE 1 (ZEBRAFISH)
209288_S_AT	CDC42EP3	CDC42 EFFECTOR PROTEIN (RHO GTPASE BINDING) 3
209354_AT	TNFRSF14	TUMOR NECROSIS FACTOR RECEPTOR SUPERFAMILY, MEMBER 14 (HERPESVIRUS ENTRY MEDIATOR)
209736_AT	SOX13	SRY (SEX DETERMINING REGION Y)-BOX 13
209872_S_AT	PKP3	PLAKOPHILIN 3
209900_S_AT	SLC16A1	SOLUTE CARRIER FAMILY 16 (MONOCARBOXYLIC ACID TRANSPORTERS), MEMBER 1
209988_S_AT	ASCL1	ACHAETE-SCUTE COMPLEX-LIKE 1 (DROSOPHILA)
210184_AT	ITGAX	INTEGRIN, ALPHA X (COMPLEMENT COMPONENT 3 RECEPTOR 4 SUBUNIT)
210513_S_AT	VEGFA	VASCULAR ENDOTHELIAL GROWTH FACTOR
210854_X_AT	SLC6A8	SOLUTE CARRIER FAMILY 6 (NEUROTRANSMITTER TRANSPORTER, CREATINE), MEMBER 8
211097_S_AT	PBX2	PRE-B-CELL LEUKEMIA TRANSCRIPTION FACTOR 2
211527_X_AT	VEGFA	VASCULAR ENDOTHELIAL GROWTH FACTOR
212375_AT	EP400	TRINUCLEOTIDE REPEAT CONTAINING 12
212467_AT	DNAJC13	DNAJ (HSP40) HOMOLOG, SUBFAMILY C, MEMBER 13
212594_AT	PDCD4	PROGRAMMED CELL DEATH 4 (NEOPLASTIC TRANSFORMATION INHIBITOR)
212739_S_AT	NME4	NON-METASTATIC CELLS 4, PROTEIN EXPRESSED IN
212837_AT	KIAA0157	KIAA0157
212878_S_AT	KLC1	KINESIN 2
213051_AT	ZC3HAV1	ZINC FINGER CCCH-TYPE, ANTIVIRAL 1
213187_X_AT	FTLL1	FERRITIN, LIGHT POLYPEPTIDE
213271_S_AT	DOPEY1	DOPEY FAMILY MEMBER 1
213451_X_AT	TNXB	TENASCIN XB
213505_S_AT	SFRS14	SPLICING FACTOR, ARGININE/SERINE-RICH 14
213756_S_AT	HSF1	HEAT SHOCK TRANSCRIPTION FACTOR 1
213757_AT	EIF5A	EUKARYOTIC TRANSLATION INITIATION FACTOR 5A
213856_AT	CD47	CD47 ANTIGEN (RH-RELATED ANTIGEN, INTEGRIN-ASSOCIATED SIGNAL TRANSDUCER)
214095_AT	SHMT2	SERINE HYDROXYMETHYLTRANSFERASE 2 (MITOCHONDRIAL)
214437_S_AT	SHMT2	SERINE HYDROXYMETHYLTRANSFERASE 2 (MITOCHONDRIAL)
214697_S_AT	ROD1	ROD1 REGULATOR OF DIFFERENTIATION 1 (S. POMBE)
215735_S_AT	TSC2	TUBEROUS SCLEROSIS 2
216017_S_AT	NAB2	NGFI-A BINDING PROTEIN 2 (EGR1 BINDING PROTEIN 2)
216080_S_AT	FADS3	FATTY ACID DESATURASE 3
216237_S_AT	MCM5	MCM5 MINICHROMOSOME MAINTENANCE DEFICIENT 5, CELL DIVISION CYCLE 46 (S. CEREVISIAE)
217693_X_AT	LOC388335	SIMILAR TO RIKEN CDNA A730055C05 GENE
217928_S_AT	SAPS3	CHROMOSOME 11 OPEN READING FRAME 23
218807_AT	VAV3	VAV 3 ONCOGENE
218887_AT	MRPL2	MITOCHONDRIAL RIBOSOMAL PROTEIN L2
218889_AT	NOC3L	NUCLEOLAR COMPLEX ASSOCIATED 3 HOMOLOG (S. CEREVISIAE)
219829_AT	ITGB1BP2	INTEGRIN BETA 1 BINDING PROTEIN (MELUSIN) 2
220116_AT	KCNN2	POTASSIUM INTERMEDIATE/SMALL CONDUCTANCE CALCIUM- ACTIVATED CHANNEL, SUBFAMILY N, MEMBER 2
221014_S_AT	RAB33B	RAB33B, MEMBER RAS ONCOGENE FAMILY
221926_S_AT	IL17RC	INTERLEUKIN 17 RECEPTOR C
222071_S_AT	SLCO4C1	HYPOTHETICAL PROTEIN PRO2176
46947_AT	GNL3L	GUANINE NUCLEOTIDE BINDING PROTEIN-LIKE 3 (NUCLEOLAR)-LIKE

**Figure 3:**
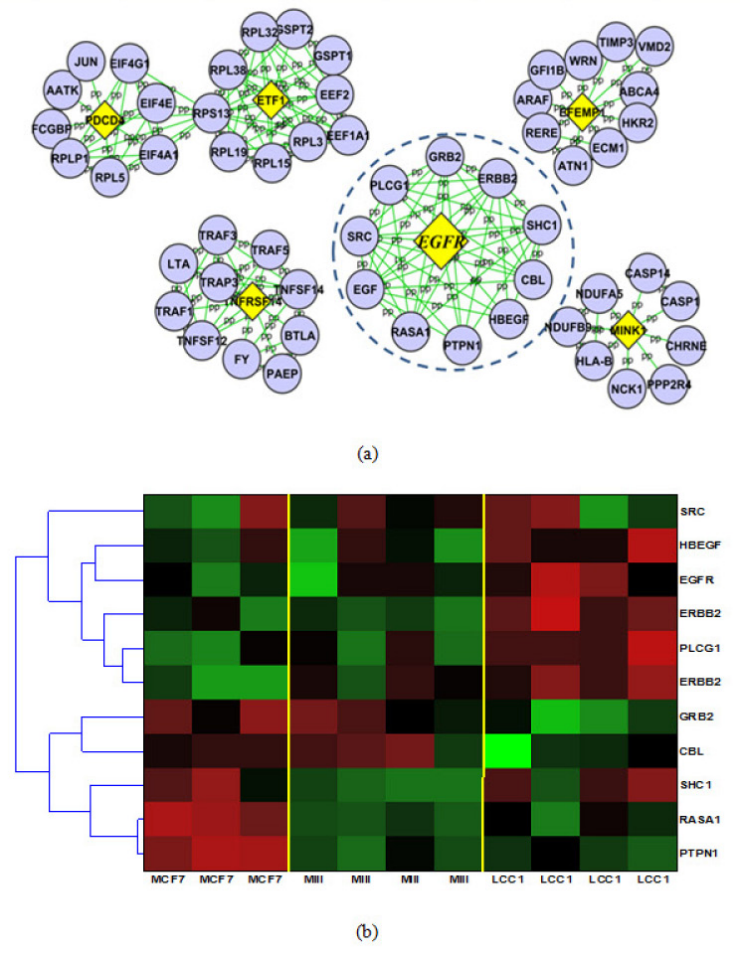
**Identified target genes of EGFR-specific transcription factor (ETF) in estrogen-induced and estrogen-deprived conditions**. (a) Yellow diamond: target genes of ETF; purple circle: direct neighbors of the target genes from protein-protein interaction (PPI) data. (b) Gene expression pattern of EGFR and its direct PPI in estrogen-deprived condition.

Notably, EGFR is among the overlapped genes, and the expression of EGFR is upregulated in LCC1 cells. We then searched the String Database to find direct neighbors of EGFR in the protein-protein interaction (PPI) network [56]. Figure 3(a) shows some of the putative ETF target genes and their PPI networks from the String Database, which notably includes EGFR and several direct neighbors of EGFR: CBL, RASA1, PTPN1, SHC1, HBEGF, SRC, ERBB2, GREB2, PLCC1. Other ETF target genes and their PPI networks can be found in the supplementary material (Fig. S8).

Figure 3(b) shows the gene expression pattern of EGFR and its direct neighbors under estrogen-deprived conditions. As we can see from the figure, the expression level of CBL was largely suppressed in the estrogen-deprived condition. Since CBL can promote the ubiquitination and degradation of activated EGFR [57], we hypothesize that EGFR expression is increased in LCC1 cells due to both the activation of ETF and the downregulation of CBL. Studies to explore these predictions are currently in progress.

Overexpression and/or activation of the ErbB receptors (ErbB1 = EGFR) may also promote proliferation, motility, adhesion, and differentiation [58]. Recent evidence has shown that increased growth factor (GF) signaling augments the ligand (estrogen)-independent activity of ER [59], which may partially explain the activity of ER (V$ER_Q6) in LCC1 cells as seen in Figure 2(b). In addition, the PLC-Gamma (PLCG1) and the JAK-STAT pathways are known to enhance the transcription of genes that regulate cell proliferation. This could contribute to the induced activity of STAT (V$STAT_Q6) (see Figure 2(b)), since one of the important signaling events activated by EGFR involves tyrosine phosphorylation of STAT. Stimulation of EGFR may induce tyrosine phosphorylation of STAT1, STAT3 and STAT5, initiating complex formation of these STATs with JAK1 and JAK2. JAKs are essential mediators of the interaction between EGFR and the STATs, which then translocate to the nucleus to stimulate gene transcription [60,61]. Importantly, we have recently shown that EGFR signaling through p130Cas and the tyrosine kinase c-Src leads to phosphorylation of STAT5B, and that this signal transduction pathway induces Tamoxifen resistance in MCF-7 breast cancer cells [62].

It is also important to validate the identified target genes by biological experiments such as other breast cancer cell line data and ChIP-on-chip experiments. While many estrogen target genes have been identified through expression microarray studies [63], the results from ChIP-on-chip experiments are not currently complete. Nonetheless, our list of ER target genes includes the following known direct targets: TFF1, GREB1 [64,65]; VAMP3 [65,66]; PRKCSH, PLEC1, NT5C2, C19ORF2, TMOD3, and FLJ11286 [65]. Furthermore, Cicatiello *et al*. have recently performed a comprehensive genome-wide analysis to investigate ERα target genes by chromatin immunoprecipitation coupled to massively parallel sequencing and expression data [67]. Comparing our gene list with their ChIP-seq and expression data showed that we find family members or isoforms of CLIC3, ELF3, RAB31, FKBP4, IGFBP4, and SLC25A19 within their ChIP-seq data. Several genes (CDT1, IGFBP5, YARS, IPO4, EPS8L1, GPR137) appear in both our target gene list and their list of genes responsive to 17β-estradiol. Currently, we are investigating several other transcription factors with biological experiments including ChIP-on-chip experiments.

To provide further statistical evidence in support of the identified ER target genes, we conducted several additional analyses including statistical significance analysis, false discovery rate (FDR) calculation, gene set enrichment analysis, and motif enrichment analysis. For these statistical analyses, we selected two recently published genomic analyses of transcription factor binding of estrogen-regulated promoters as a benchmark [63,67]; we acknowledge the incompleteness of ChIP-on-chip data for ER target genes across multiple cellualr contexts. Firstly, a statistically significant enrichment of ER target genes can be observed in our ER target gene list, as supported by the statistical significance (*p-*value = 3.59×10^-06^) calulated based on the assumption of a hyper-geometric distribution in a comparison with the ChIP-on-chip benchmark target genes. A low false positive rate is evident (FDR = 9.72×10^-09^) for the ER target gene list identified by mSD.

To calculate the FDR, we first ranked all the genes according to their computed binding strength in matrix **S **to $ER_Q6 binding site; we then selected a 'negative' set of genes with no binding connection with $ER_Q6 in position weight matrix (PWM) to form a null distribution of the binding strength. As in the mSD approach, we assumed that the binding strength of target genes regulated by a transcription factor roughly follows a Gamma distribution, since most transcription factors likely regulate relatively few target genes. Thus, we calculated the *p*-value for each gene by selecting the strongest binding strength when compared with those obtained from the null distribution. To properly determine a cut-off threshold of the binding strength, we also controlled the FDR for multiple tests based on the total number of genes in the experiments [68]. We used the Benjamini-Hochberg procedure [69] to compute the false discovery rate as follows. Letting *p_k _*represent the corrected *p-*value computed for gene *k*, *r_k _*the rank of gene *k *sorted by the *p*-values, and *G *the total number of genes in the experiment, we calculated the false discovery rate for gene *k *as *FDR_k _*=*Gp_k_*/*r_k_*. For our identified ER taget gene list, we obtaned a low FDR (FDR = 9.72×10^-09^) corresponding to a binding strength cutoff of 0.7.

We also used a Kolmogorov-Smirnov (KS) test to evaluate the enrichment of ER target genes [70]. We first ordered all the genes in our experiments according to their computed binding strength in matrix **S**. We then formed the distribution of the target gene set within this ordered list by the KS nonparametric rank statistic as described below [70]. First, we denote *n *the total number of genes in the ordered ER target list, *x *the number of overlapped genes between our inferred target genes and the ChIP-on-chip benchmark data, and *y *the number of non-ovarlepped genes. Second, we let *V*(*i*) = *y*, if gene *i *is included in the overlapped genes; *V*(*i*) = -*x*, if not; note that we have  from this configuration. Finally, we define the KS rank statistic as follows:  to conduct this statistical test based on a permutation test [71]. For our ER target gene list, the KS score (KS_score = 208) is significantly higher than the scores in the null distribution based on 10,000 randomly selected gene sets of the same size as the inferred ER target genes (with a statistical significance of *p*-value = 0.0099; see Fig. S9 in the supplementary material).

We evaluated the enrichment of ER binding sites in the promoters of target genes identified by the mSD approach using TRANSFAC [29]. A motif enrichment analysis procedure was used based on a permutation test [72], which can be summarized as follows. Given a gene set *S *extracted by any computational method such as the mSD approach, a statistic to measure the enrichment of a specific motif *f *is defined as , where *m *is the motif binding score as defined by both matrix similarity score and core similarity score [29,72]. To calculate the statistical significance (*p*-value), we need to form a null distribution. The null hypothesis is that the gene set is randomly generated from the gene population and there is no significant enrichment of the motif *f*. We randomly select gene sets with same size of *S *from the baseline gene population, and repeat *B *times to generate the corresponding null statistic enrichment score , for *b *= 1,..., B. The null hypothesis distribution is assumed to be symmetric in this study. The *p*-value can be obtained for each gene set by calculating the probability that a null gene set has a larger statistic than the observed statistic. Mathematically, the p-value can be calculated by

**Figure 4:**
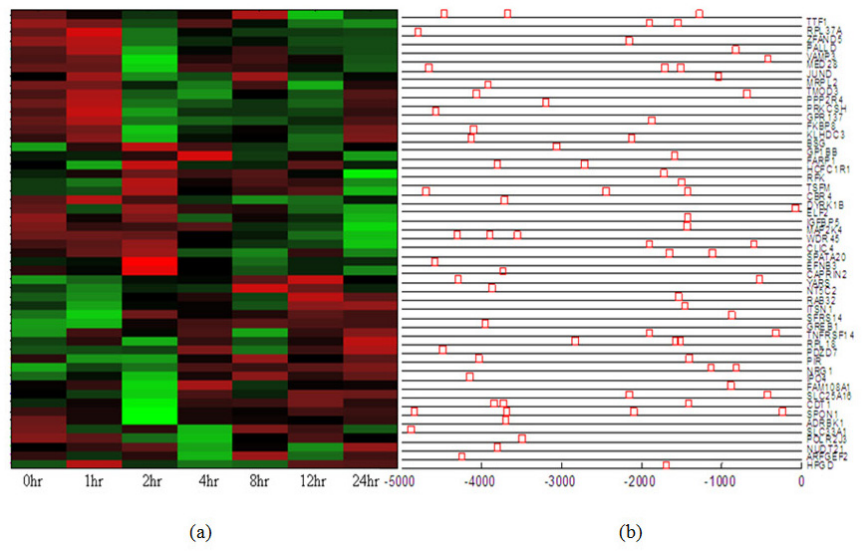
**Expression pattern and $ER_Q6 binding distribution of ER target genes in estrogen-induced condition (MCF-7 cell line)**. (a) The identified ER target genes show a consistent gene expression pattern of either being early-induced (≤ 4 hours) or late-induced (4 - 24 hours) by estrogen. (b) 52 out of 68 ER target genes have at least one $ER_Q6 binding site.

. By comparing our identified ER target gene list to a randomly selected gene list (repeated 10,000 times), we clearly demonstrated a statistically significant enrichment of ER binding site in the identified ER target genes (*p*-value < 10^-04^). The distribution of $ER_Q6 binding site among the identified ER target genes is shown in Figure 4, along with the gene expression pattern of these ER target genes in MCF-7 cell line data.

## Conclusions

Traditional clustering methods have been widely used for gene module identification by searching for similar patterns in gene expression data. Clustering methods on gene expression data alone can only provide co-expressed gene modules. The expression pattern of genes in the same cluster may be correlated for reasons other than co-regulation. To identify gene regulatory modules, it is important to incorporate transcription factor binding information based either on ChIP-on-chip data or on motif information. The proposed method, namely, motif-guided sparse decomposition (mSD), is an integrated approach to combine gene expression data and binding information for regulatory module identification.

The main challenge is that the level of noise is high in both of the data types to be integrated. If a simple integration strategy is used, the method will result in many false positive target genes due to noise. Two strategies were developed in our mSD approach to mitigate the effects of noise impact on target gene identification. Firstly, an affinity propagation (AP) clustering method [22] is used to estimate transcription factor activity by clustering gene expression data in conjunction with binding information. Secondly, a sparse component analysis (SCA) method [19] is applied to estimate regulation strength by exploiting the constraint that most genes are regulated by only a few transcription factors. Since a gene cluster formed using an AP method reflects a similar pattern (from the gene expression data) and a shared regulator (from the binding information), the transcription factor activity (TFA) estimated from the cluster is a better starting point for regulatory module identification. Using a SCA method and the improved TFA estimates further refines the gene cluster by estimating the regulation strength of a particular transcription factor.

The mSD approach has been developed and implemented as follows. Binding motif information is initially used to define potential target genes, providing prior knowledge of the regulatory network topology. A sparse latent variable model is then used to integrate gene expression data and identify which of the potential target genes are actually activated by transcription factors. The mSD approach was implemented as a two-step algorithm to perform (1) transcription factor activity estimation, and (2) regulation strength estimation. In the first step, we start to integrate binding motif information and gene expression data to identify co-regulated gene clusters. A motif-guided gene cluster method was developed and used to find the gene clusters, based on a joint similarity measure from both gene expression data and motif information. To limit the impact of noise on gene clustering performance, the contribution of each data type to clustering is quantified. The optimal trade-off between data sources can then be determined by minimizing a cost function taking into account the frequency of motif occupancy and non-uniformity of expression pattern. Subsequently, we use a sparse decomposition method for regulation strength estimation.

Unlike the NCA method [16] that assumes the network topology derived from ChIP-on-chip data or motif information is known without error, we consider both network configuration and connection strength estimation as integrative components of the decomposition method. The use of prior knowledge of binding motif-information provides a solid starting point. As in Sabatti's work [17], we also incorporate a sparse constraint to achieve a biologically meaningful representation of regulatory networks. The experimental results on synthetic and real yeast data have demonstrated that our method can effectively identify the target genes of transcription factors. The application of mSD to breast cancer cell line data further revealed condition-specific regulatory modules associated with estrogen signaling and action in breast cancer, which are consistent with known gene functions in this cellular context.

The current work represents an important step toward integrating available biological information for reconstructing complex biological networks. This goal will be better accomplished by incorporating an analysis of the synergistic effect of regulators into the proposed method. Combinatorial analysis may help discover the complex interplay between different regulators in order to assemble a complete map of regulatory networks for complex biological systems.

## Authors' contributions

TG and JX formulated the problem and developed the theoretical framework of the algorithm. TG carried out the development and implementation of the algorithm. LC, RBR, HL, YW, EPH and RC provided technical and biological support to the project. All authors participated in the writing of the manuscript, and have read and approved the manuscript.

## Supplementary Material

Additional file 1 **Supplementary material**. Supplementary material includes supplementary method, tables and figures. Click here for file

Additional file 2 **Target genes of four transcript ion factors (i.e., AP-1, ER, STAT and NFκB), respectively**. The target gene lists can be found in 'Target_Genes_TFs.xls'. Click here for file
