# Supplementary material for: Motif-guided sparse decomposition of gene expression data for regulatory module identification
Source: BMC Bioinformatics. 2011 Mar 22;12:82. doi: 10.1186/1471-2105-12-82 (PMC3072956; doi:10.1186/1471-2105-12-82)
Supplement: Additional file 1 — Supplementary material. Supplementary material includes supplementary method, tables and figures. [file 1471-2105-12-82-S1.PDF]

Supplementary Material

Motif-guided Sparse Decomposition of Gene Expression Data for Regulatory Module Identification

Ting Gong[1], Jianhua Xuan[1*], Li Chen[1], Rebecca B. Riggins[2], Huai Li[3], Eric P. Hoffman[4], Robert Clarke[2], and Yue Wang[1]

[1]Department of Electrical and Computer Engineering, Virginia Polytechnic Institute and State University, Arlington, VA 22203, USA

[2]Lombardi Comprehensive Cancer Center and Department of Oncology, Physiology and Biophysics, Georgetown University, Washington, DC 20057, USA

[3]Bioinformatics Unit, RRB, National Institute on Aging, National Institutes of Health, Baltimore, MD 21224, USA

[4]Research Center for Genetic Medicine, Children's National Medical Center, Washington, DC 20010, USA.

I. Supplementary Method

S1 Transcription factor activity estimation

S1.1 Identifiability of motif-guided Sparse Decomposition (mSD)

In order to reliably estimate transcription factor activity (**A**), the sparsity property of the regulation strength matrix **S** is very important. In fact, the following theorem is the key to obtaining a reliable estimation of **A** [1].

Theorem: (Identifiability conditions - locally very sparse representation): Assume that the number of transcription binding sites (TFBSs) is unknown and the following two conditions are met:

1) each TFBS has at least two strictly well-grounded points (sWGPs), which means that for each index $i = 1, \cdots, n$, there are at least two columns of **S**: $\mathbf{s}(:, j_1)$ and $\mathbf{s}(:, j_2)$ that have nonzero elements only in position i (i.e., each TFBS is uniquely present at

least twice);

2) for any $\mathbf{x}(:,k) \neq c\mathbf{x}(:,q)$, any $c \in \mathbf{R}$, $k, q = 1, \cdots, N$ and $k \neq q$, $\mathbf{s}(:, k)$ has more than one nonzero element.

Then, **A** is uniquely determined by **X** except for left multiplication with a permutation and scaling matrix. For proofs of the theorem we refer to [1].

Under this identifiability condition, we propose to use a motif-guided clustering method to find representative columns of **X** for an estimation of **A**.

S1.2 screening transcription factor binding motifs

we do not perform motif discovery as part of our learning procedure, but rather assume that we have a list of motifs for putative transcription factor binding sites (TFBSs) by searching a database of regulatory elements such as TRANSFAC [2]. Our learning algorithm only inputs validated TFBSs that allow for a straight-forward biological interpretation, which facilitates biologists to decipher the function of genes being regulated under a given experimental condition. In this paper, all human promoter DNA sequences were obtained from the UCSC Genome database [4]; in particular, upstream 5,000 bp from the transcription start site (TSS) was obtained. Cautions are also needed when considering the distance to TSS for binding strength in high eukaryotes, especially human genome. In high eukaryotes, especially human genome, gene promoter region is defined in a relative large range, e.g., upstream 1,000 bp, 2,000 bp or 5,000 bp from TSS to downstream hundreds or thousands base

pair. For example, a recent genome-wide ChIP-on-chip study shows that only 4% of estrogen receptor binding sites are mapped to 1,000 bp promoter-proximal regions [3]. Many sites are located in regions of 1,000 bp to 5,000 bp away from TSS. We thus used distance from TSS 5,000 bp to define the initial motif binding strength based on motif score and number of occurrences. With all vertebrate position weight matrices (PWMs) provided by the TRANSFAC 11.1 Professional Database [2], Match™ [5] algorithm was used to generate a gene-motif binding strength matrix with the cut offs that minimize the false-positive rate.

S2 Regulation Strength estimation by sparse decomposition

For the sparse decomposition of gene expression data, the solution set of $\mathbf{x} = \mathbf{A}\mathbf{s}$, in variable **s**, defines an affine set in $\mathbf{R}^n$. By minimizing the cost function $\sum_{t=1}^{q} s_{tg}^2$ (with assumed q "inactive" TFs), we suppress the regulation strength of "inactive" TFs while leaving the regulation strength of "active" TFs to change freely in the active subspace in order to fulfill the constraint condition $\mathbf{x} = \mathbf{A}\mathbf{s}$. This may also be viewed as a form of projection into the active subspace [6]. In fact, the cost function can be reformulated into a quadratic form: $\boldsymbol{f}(\mathbf{s}) = \mathbf{s}^T \mathbf{H}\mathbf{s}$ with $\mathbf{H} = \begin{pmatrix} \mathbf{I}_q & \mathbf{0} \\ \mathbf{0} & \mathbf{0} \end{pmatrix}$ where $\mathbf{I}_q$ is the $q \times q$ identity matrix. When the cost function $\boldsymbol{f}$(s) is strictly convex for all feasible points, it has a unique local minimum that is also the global minimum. A sufficient condition to guarantee the strict convexity of $\boldsymbol{f}$(s) is for **H** to be positive definite [7]. The projection into active subspace finally leads to an elegant solution to our sparse

decomposition problem, i.e., the solution to the following Karush-Kuhn-Tucker (KKT) system [6]:

$$\begin{pmatrix} \mathbf{H} & \mathbf{A}^T \\ \mathbf{A} & \mathbf{0} \end{pmatrix} \begin{pmatrix} \mathbf{s} \\ \lambda \end{pmatrix} = \begin{pmatrix} \mathbf{0} \\ \mathbf{x} \end{pmatrix}, \qquad \text{(S1)}$$

where λ is the $n \times 1$ vector of Lagrange multipliers.

II. Supplementary Tables

Table S1 - AUC Comparison of mSD, SD and FastNCA methods for 11 transcription factors in yeast synthetic data

Cut-off *p*-value	Method	ARG80	DAL82	GCN4	GCR2	HAP1	MIG1	RGT1	RTG1	RTG3	STE12	XBP1	Averaged AUC over TFs
***p* = 0.1**	**mSD**	0.5892	0.7057	0.8204	0.7827	0.9238	0.8390	0.8180	0.3281	0.4890	0.7487	0.8318	**0.7160**
	**FastNCA**	0.4634	0.4466	0.8129	0.6807	0.1435	0.5531	0.3873	0.8297	0.8612	0.5318	0.5672	0.5707
	**SD**	0.4984	0.9290	0.8730	0.6485	0.5226	0.9022	0.7246	0.8180	0.4483	0.5055	0.7326	0.6912
***p* = 0.05**	**mSD**	0.5311	0.6859	0.8344	0.7811	0.9335	0.8251	0.8222	0.7382	0.8454	0.7325	0.8497	**0.7799**
	**FastNCA**	0.4554	0.4418	0.8099	0.4196	0.0795	0.5234	0.7175	0.8454	0.8517	0.4960	0.8402	0.5891
	**SD**	0.5916	0.7607	0.8784	0.6341	0.4858	0.8927	0.7422	0.8688	0.4704	0.5211	0.7233	0.6881
***p* = 0.01**	**mSD**	0.7779	0.7105	0.8154	0.7938	0.9738	0.8136	0.9058	0.7413	0.8265	0.7234	0.7439	**0.8024**
	**FastNCA**	0.4618	0.4493	0.7974	0.4276	0.0773	0.4725	0.3831	0.8612	0.8644	0.4700	0.8370	0.5547
	**SD**	0.5255	0.7660	0.8613	0.6180	0.5304	0.8801	0.7418	0.8730	0.4735	0.4836	0.7279	0.6801

III. Supplementary Figures

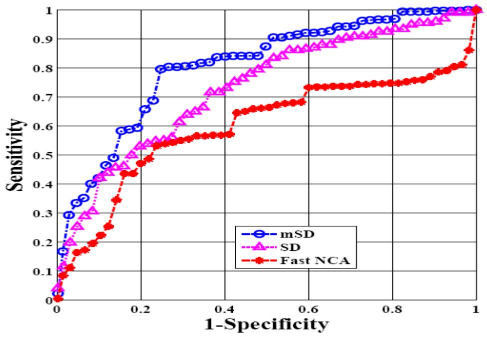

(a)

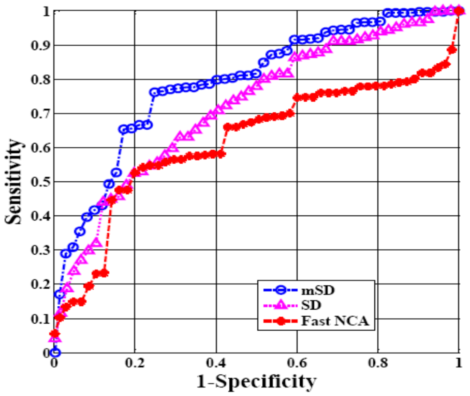

(b)

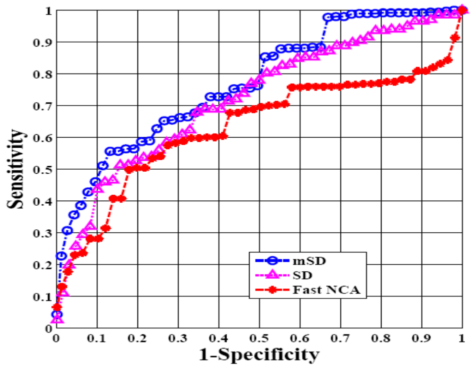

(c)

Figure S1 - Comparison of Receiver Operator Characteristic (ROC) curves for mSD and other methods (i.e., SD and FastNCA) on simulation data

Three different cut-off p-values are used to compare the methods for their tolerance in the false positive rate of binding information: (a) cut-off *p*-value = 0.05; (b) cut-off *p*-value = 0.01; (c) cut-off *p*-value = 0.1.

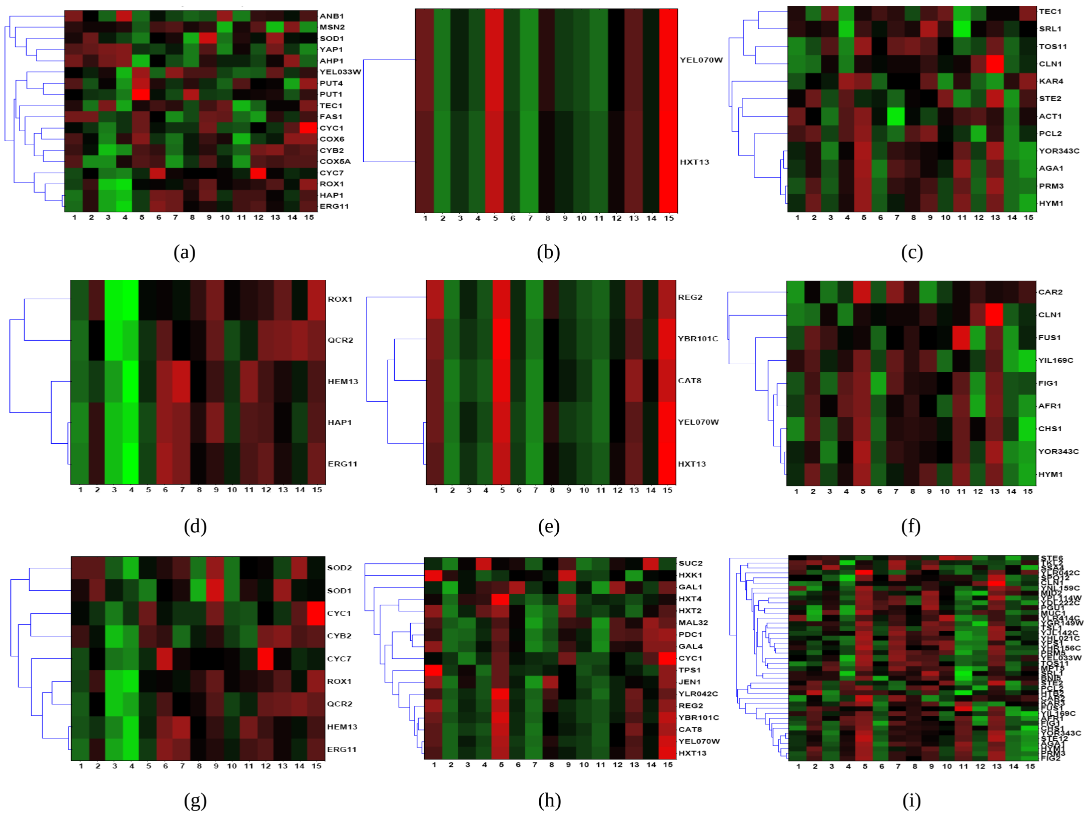

Figure S2 - Gene clusters identified as co-regulated by HAP1 (left), MIG1 (middle) and STE12 (right), respectively

The first row: initial clusters from ChIP-on-chip data for HAP1 (a), MIG1 (b) and STE12 (c), respectively; the second row: identified target genes of HAP1 (d), MIG1 (e) and STE12 (f), respectively; the third row: the ground truth of target genes regulated by HAP1 (g), MIG1 (h) and STE12 (i).

By integrating known TF binding site information and gene expression data with the mSD approach, we can identify three different scenarios of co-regulated genes that are not only co-expressed but also share common regulatory elements in a condition-dependent way. The three scenarios are termed as "*condition-enabled*", "*condition-expanded*" and "*condition-combined*" in this paper, which are illustrated in Fig. S2 and discussed as follows:

Scenario 1 - "*condition-enabled*": in this scenario, the TFs regulate some of their

target genes in one condition but not in others. For example, the initial cluster associated with HAP1 obtained from ChIP-on-chip data alone shows a complex pattern of gene expression (Fig. S2(a)); the mSD approach selected a subset of genes in the initial cluster by consulting with the expression data, showing a much coherent expression pattern (Fig. S2(d)). Comparing with the ground truth from the simulation, the genes selected by mSD correspond to a major portion of the regulated genes by HAP1 (Fig. S2(g)). Since the experimental condition of the available ChIP-on-chip data is not consistent with that of the gene expression data, not all the initial genes from ChIP-on-chip data are activated or transcribed. Instead, the genes are regulated in a "condition-enabled" way, i.e., only a subset of genes being actually activated and transcribed with a coherent pattern of gene expression. This scenario indicates that the binding of HAP1 to its target genes might be influenced by different experimental conditions.

Scenario 2 - "*condition-expanded*": in this second scenario, the target genes in one condition are further expanded to include more target genes in another condition. For example, the MIG1 ChIP-chip data give us only two target genes, YEL070W and HXT13, when the cut-off *p*-value is relatively small (cut-off p-value = 0.01) (Fig. S2(b)). With the help of gene expression data, the mSD approach can help find more target genes (Fig. S2(e) that are actually included in the simulation (i.e., the ground truth) (Fig. S2(h)). As we can see from the figure, the mSD approach selected a subset of genes showing a highly coherent pattern of gene expression with expanded support from binding information (noting that the actual cut-off p-value used to generate the

gene expression data is relatively large).

Scenario 3 -“*condition-combined*”: as the third scenario, the target genes of a TF were identified as combined genes from different conditions. For each TF, we allocated the target genes by gathering genes with similar expression patterns and shared binding site(s). For example, the target genes of STE12 are shown in Fig. S2. As we can see from the figure, the initial cluster associated with STE12 obtained from ChIP-on-chip data (Fig. S2(c)) shows a relatively simple expression pattern, but from the gene expression data, the actual target genes of STE12 shows a much complex expression pattern (Fig. S2(i)). The complex expression pattern is supported by the following biological studies. STE12 was reported to participate in the cell wall integrity signaling pathway [8], and to constitute a coordinated group with other TFs regulating genes involved in cell cycle control or regulation of telomere maintenance [9]. The mSD approach selected a combined subset of genes as shown in Fig. S2(f); in addition to the genes supported by both data sources, some genes are backed up by ChIP-on-chip data and the others by gene expression data. This scenario demonstrates that the mSD approach can obtain “condition-combined” target genes from both gene expression data and binding information.

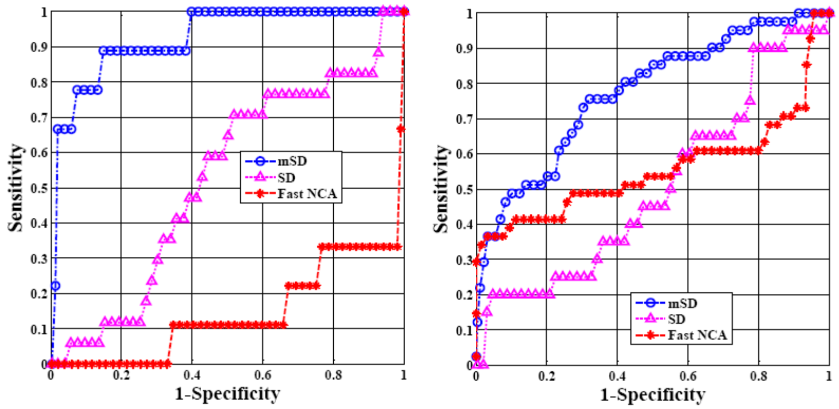

(a) cut-off *p*-value = 0.1

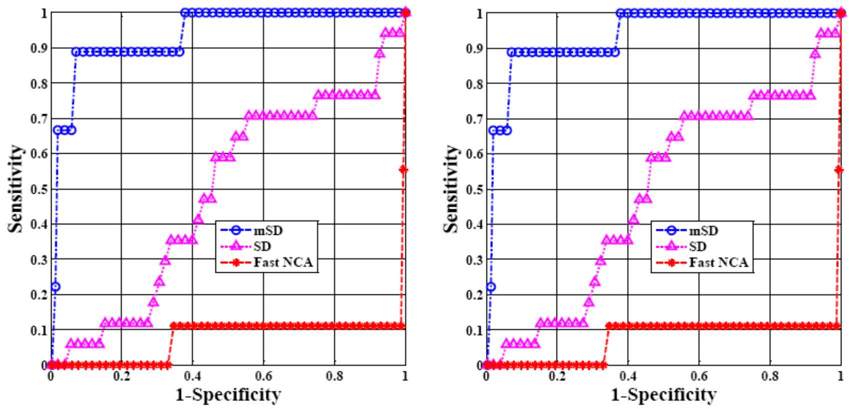

(b) cut-off *p*-value = 0.05

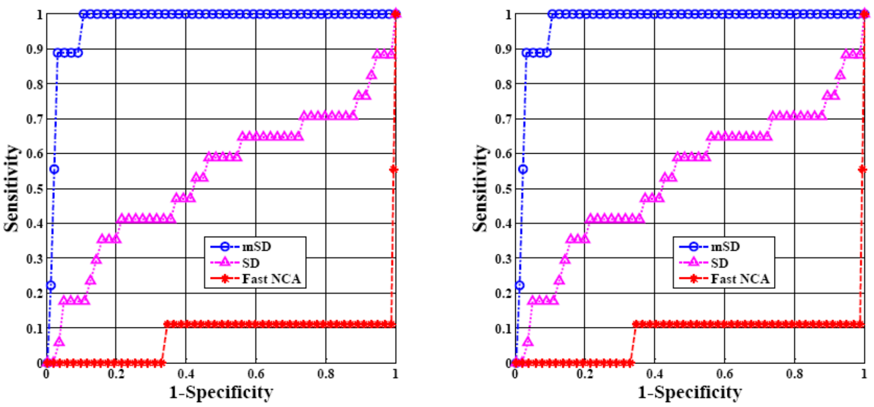

(c) cut-off *p*-value = 0.01

Figure S3 - Performance comparison of mSD, SD and FastNCA methods - ROC curves for the identified regulatory modules of HAP1 (left) and STE12 (right), respectively

Three different cut-off p-values (0.1, 0.05 and 0.01) have been applied to ChIP-on-chip data for investigating the noise impact on the performance.

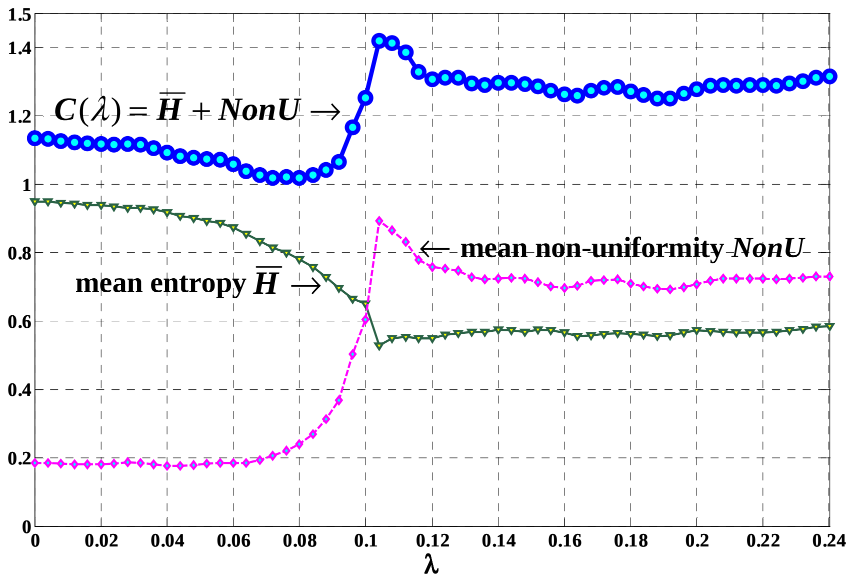

Figure S4 - Determination of the trade-off parameter λ for yeast cell cycle data

Dark-green triangle: mean entropy of motif occupancy; magenta diamond: mean non-uniformity of gene expression pattern; blue circle: $C(\lambda)$ that adds up mean entropy of motif occupancy and mean non-uniformity of gene expression pattern.

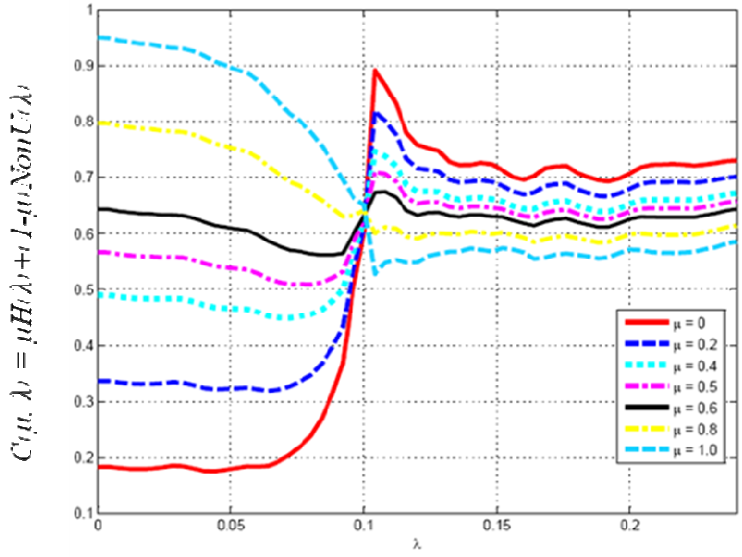

Figure S5 - The modified cost function, *C*(*μ, λ*) = *μH*(*λ*)+(*1-μ*)*NonU*(*λ*), for the yeast cell cycle study

For this study, we can see from the figure that with the new cost function, the determination of parameter λ is not sensitive to parameter μ when $0.4 \leq \mu \leq 0.6$. When μ is out of the above-mentioned range, we can see that the cost function does not give rise to a clear U-shaped curve. In particular, when $\mu > 0.6$ the cost function is largely bised to motif occupancy (measure by $H(\lambda)$), while ignoring expression pattern (measure by $NonU(\lambda)$); conversely, when $\mu < 0.4$, the cost function is biased to expression pattern while ignoring the motif occupancy information.

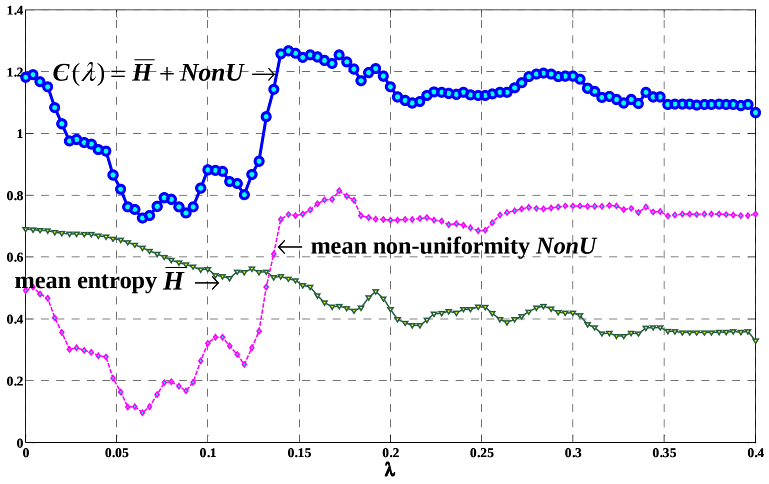

(a)

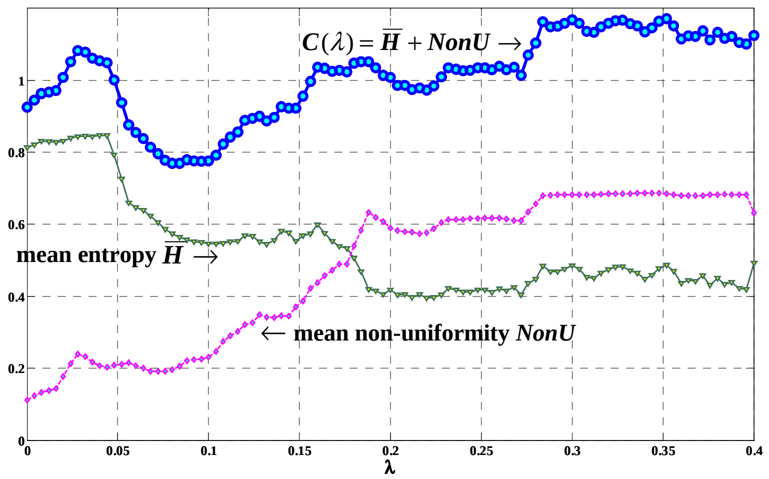

(b)

Figure S6 - Determination of the trade-off parameter λ for breast cancer cell line data: (a) estrogen-induced condition and (b) estrogen-deprived condition

Dark-green triangle: mean entropy of motif occupancy; magenta diamond: mean non-uniformity of gene expression pattern; blue circle: $C(\lambda)$ that adds up mean entropy of motif occupancy and mean non-uniformity of gene expression pattern.

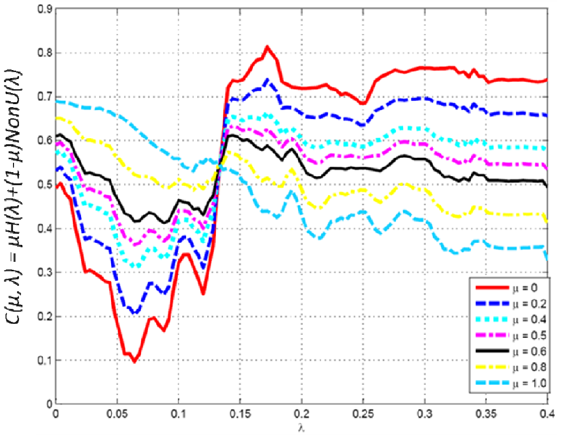

(a)

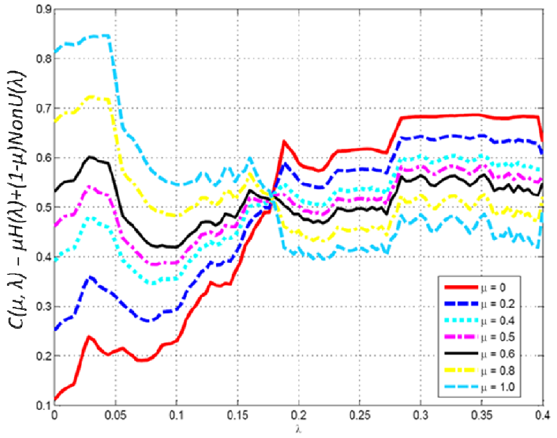

(b)

Figure S7. The new cost function, $C(\mu, \lambda) = \mu H(\lambda)+(1-\mu)NonU(\lambda)$, for the breast cancer study: (a) estrogen-induced condition and (b) estrogen-deprived condition

For the estrogen-induced condition, it can be observed that with the new cost function, the determination of parameter λ is not sensitive to parameter μ when $0\leq\mu\leq0.6$; for the estrogen-deprived condition, the determination of parameter λ is not sensitive to parameter μ when $0.2\leq\mu\leq0.6$. However, when μ is out of the above-mentioned range, particularly, when $\mu>0.6$, we can clearly see that the cost function does not give rise

to a clear U-shaped curve, since it is largely bised to motif occupancy (measure by $H(\lambda)$), while ignoring expression pattern (measure by $NonU(\lambda)$). Therefore, we cautiously conclude that paramter μ is an important paramter to be specified for a specific study, although the obtained results from our breast cancer study are not sensitive to paramter μ when $0.2 \leq \mu \leq 0.6$.

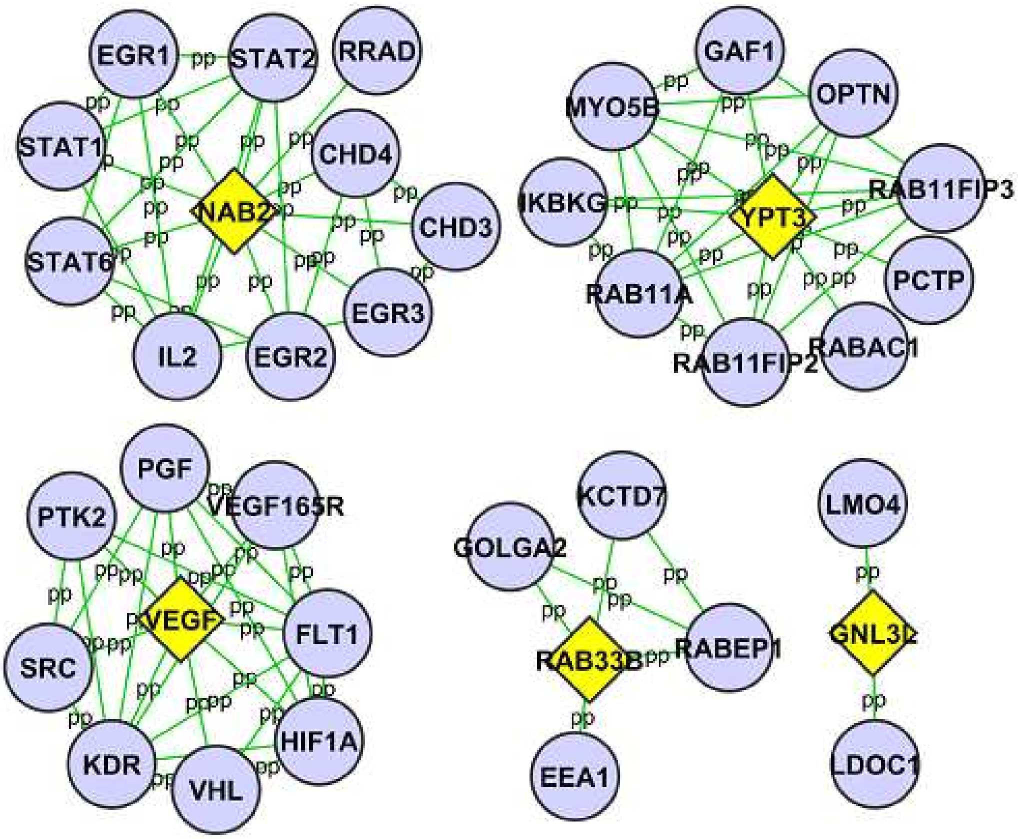

Figure S8 - More PPI subnetworks of target genes of ETF identified in estrogen-induced and estrogen-deprived conditions

Yellow diamond: target genes of ETF; purple circle: direct neighbors of the target genes as obtained from protein-protein interaction data.

In Fig. S8, we found several proteins such as YPT3, Rab33B related to Ras signal transduction pathway. Since Ras-dependent events appear to be activated as a consequence of EGFR mutations in cancer cells [10], it is possible that the aberrant function of Ras-related proteins may contribute to breast cancer development [11] by a network of proto-oncogene proteins controlling diverse signaling events that regulate cell growth and differentiation defined in Ras signal transduction pathway.

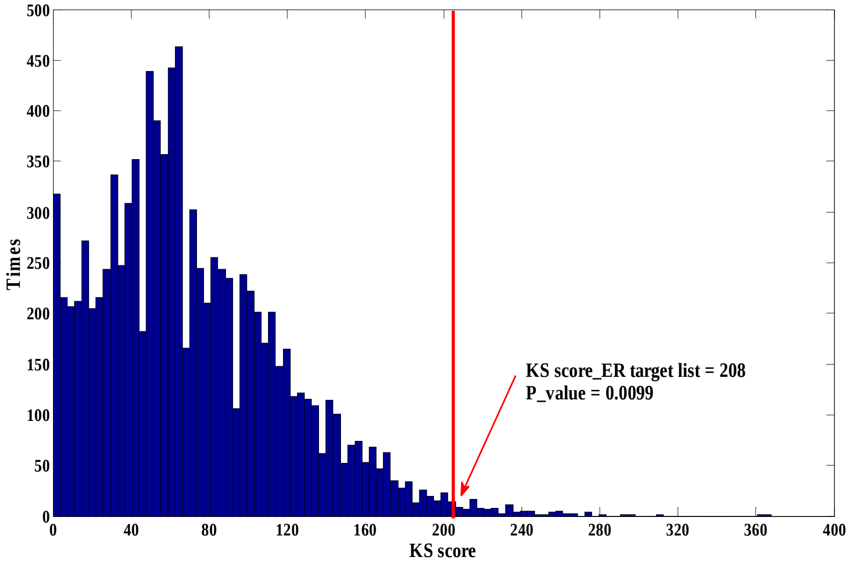

Figure S9 - The distribution of Kolmogorov-Smirnov (KS) rank statistic

We selected gene sets with the same size of the inferred ER target gene list from the background gene population, and repeated 10,000 times to generate the corresponding null distribution of the Kolmogorov-Smirnov (KS) rank statistic. The KS score of our inferred ER target gene set is indicated as the red line (at KS score = 208) in the figure.

References

1. Georgiev P, Theis F, Cichocki A: **Sparse component analysis and blind source separation of underdetermined mixtures**. *Neural Networks, IEEE Transactions on* 2005, **16**(4):992-996.
2. Matys V, Kel-Margoulis OV, Fricke E, Liebich I, Land S, Barre-Dirrie A, Reuter I, Chekmenev D, Krull M, Hornischer K *et al*: **TRANSFAC(R) and its module TRANSCompel(R): transcriptional gene regulation in eukaryotes**. *Nucl Acids Res* 2006, **34**(suppl_1):D108-110.
3. Carroll JS, Meyer CA, Song J, Li W, Geistlinger TR, Eeckhoute J, Brodsky AS, Keeton EK, Fertuck KC, Hall GF *et al*: **Genome-wide analysis of estrogen receptor binding sites**. *Nat Genet* 2006, **38**(11):1289-1297.
4. Karolchik D, Baertsch R, Diekhans M, Furey TS, Hinrichs A, Lu YT, Roskin KM, Schwartz M, Sugnet CW, Thomas DJ *et al*: **The UCSC Genome Browser Database**. *Nucleic Acids Res* 2003, **31**(1):51-54.
5. Kel AE, Gossling E, Reuter I, Cheremushkin E, Kel-Margoulis OV, Wingender E: **MATCHTM: a tool for searching transcription factor binding sites in DNA sequences**. *Nucl Acids Res* 2003, **31**(13):3576-3579.
6. Arash Ali A, Massoud B-Z, Christian J: **A Fast Method for Sparse Component Analysis Based on Iterative Detection-Estimation**. *AIP Conference Proceedings* 2006, **872**(1):123-130.
7. Nocedal J, Wright S: **Numerical Optimization**, 2nd ed. edn; 2006.
8. Garcia R, Bermejo C, Grau C, Perez R, Rodriguez-Pena JM, Francois J, Nombela C,

Arroyo J: **The Global Transcriptional Response to Transient Cell Wall Damage in Saccharomyces cerevisiae and Its Regulation by the Cell Integrity Signaling Pathway**. *J Biol Chem* 2004, **279**(15):15183-15195.

9. Lee H-G, Lee H-S, Jeon S-H, Chung T-H, Lim Y-S, Huh W-K: **High-resolution analysis of condition-specific regulatory modules in Saccharomyces cerevisiae**. *Genome Biology* 2008, **9**:R2.

10. Haley JD, Gullick WJ: **EGFR Signaling Networks in Cancer Therapy** Humana Press; 2009.

11. Clark GJ, Der CJ: **Aberrant function of the Ras signal transduction pathway in human breast cancer**. *Breast Cancer Research and Treatment* 1995, **35**(1).
